# Cross-Talk and Information Transfer in Mammalian and Bacterial Signaling

**DOI:** 10.1371/journal.pone.0034488

**Published:** 2012-04-18

**Authors:** Samanthe M. Lyons, Ashok Prasad

**Affiliations:** 1 School of Biomedical Engineering, Colorado State University, Fort Collins, Colorado, United States of America; 2 Chemical and Biological Engineering, Colorado State University, Fort Collins, Colorado, United States of America; National Cancer Center, Japan

## Abstract

In mammalian and bacterial cells simple phosphorylation circuits play an important role in signaling. Bacteria have hundreds of two-component signaling systems that involve phosphotransfer between a receptor and a response regulator. In mammalian cells a similar pathway is the TGF-beta pathway, where extracellular TGF-beta ligands activate cell surface receptors that phosphorylate Smad proteins, which in turn activate many genes. In TGF-beta signaling the multiplicity of ligands begs the question as to whether cells can distinguish signals coming from different ligands, but transduced through a small set of Smads. Here we use information theory with stochastic simulations of networks to address this question. We find that when signals are transduced through only one Smad, the cell cannot distinguish between different levels of the external ligands. Increasing the number of Smads from one to two significantly improves information transmission as well as the ability to discriminate between ligands. Surprisingly, both total information transmitted and the capacity to discriminate between ligands are quite insensitive to high levels of cross-talk between the two Smads. Robustness against cross-talk requires that the average amplitude of the signals are large. We find that smaller systems, as exemplified by some two-component systems in bacteria, are significantly much less robust against cross-talk. For such system sizes phosphotransfer is also less robust against cross-talk than phosphorylation. This suggests that mammalian signal transduction can tolerate a high amount of cross-talk without degrading information content. This may have played a role in the evolution of new functionalities from small mutations in signaling pathways, allowed for the development of cross-regulation and led to increased overall robustness due to redundancy in signaling pathways. On the other hand the lack of cross-regulation observed in many bacterial two-component systems may partly be due to the loss of information content due to cross-talk.

## Introduction

Phosphorylation reactions make up a large part of signal transduction processes. However there are many different topologies of phosphorylation-based signal transduction systems. In mammalian cells one of the simplest signal transduction networks is TGF-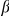 signaling. TGF-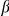 family members constitute a large class of related secreted polypeptides that are very important, especially during growth and development processes [1]. These proteins have been classified into several sub-families, of which the TGF-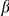 subfamily of TGF-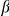
's 1, 2 and 3 and the BMP sub-family, consisting of BMPs 2, 4, 5, 6, 8 and 9, is the most important. TGF-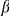 family proteins signal through trans-membrane serine/threonine kinases known as Type I and Type II receptors. The TGF-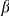 subfamily promotes the formation of a Type I/Type II complex after binding, while the BMP subfamily is believed to bind to a preformed complex of Type I/Type II receptors [2]. In either case, binding leads to phosphorylation of the cytoplasmic tail of the Type I receptor by the Type II receptor. The phosphorylated Type I receptor then recruits a subfamily of Smad proteins, called receptor Smads (or RSmads), that are phosphorylated by the Type I receptor. The 5 RSmads are the only known direct effectors of the TGF-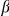 family of proteins and of them, Smad 1, 5 and 8 are preferentially used by BMP sub-family signaling and Smad 2 and 3 by the TGF-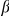 subfamily. Smad 4 is called a CoSmad and it binds with the phosphorylated RSmads and facilitates nuclear import. Smads 6 and 7 are a class of Smads called inhibitory Smads, or ISmads, and they negatively regulate Smad signaling [1].

Since the TGF-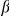 proteins are involved in diverse cellular and developmental processes, and the many proteins play non-redundant functions in vivo, the simple topology of the signaling pathway begs the question as to how specificity of signaling is maintained. The BMP subfamily, for example, can be divided further into two smaller families based on amino acid similarity, one containing BMP2 and BMP4 and the other the remaining BMPs. There is significant amino acid similarity within the BMP subfamily members, and even between subfamilies, but evidence suggests that they play non-redundant roles in vivo [3], [4], suggesting that the cell must be able to distinguish between the signals emanating from different BMP ligands.

Ligands in the extra-cellular space appear to preferentially bind different classes of receptors, and in particular it has been shown that BMPs 2/4 preferentially utilize the Type I receptor BMPR1A and the Type II receptor BMPR2 while BMPs 6/7 preferentially utilize ACVR1A and ACVR2A [5], but as far as is known they both signal through the same set of receptor Smads. It is therefore not clear whether the cell can distinguish between different signals carried by the same Smad given noisy chemical reactions. Since the number of TGF-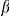 family ligands are much larger than the number of receptor Smads, it is also not clear whether the cell can discriminate between signals carried by different Smads in the presence of significant cross-talk between them.

Other signaling pathways share a similar topology as the TGF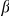 – BMP – Smad pathway discussed here, such as the Jak-Stat pathway [6]. In fact they constitute what can be called the *bow-tie* network topology [7], wherein a large number of ligands activate a large number of genes through a smaller number of intermediary proteins. Thus cross-talk is hardwired into the structure of many mammalian signaling pathways.

In bacterial cells, a similar phosphorylation-based signal transduction motif is the two-component system. Here a cell surface receptor, usually a histidine kinase (HK), autophosphorylates when bound by a cognate ligand. The phosphate group is transferred to another protein called the response regulator (RR) which now becomes a transcription factor. One key difference between the bacterial and the mammalian systems is that the cell surface receptor in the latter is an enzyme for phosphorylation of the receptor Smad that carries the signal to the nucleus, and therefore one receptor molecule can phosphorylate many receptor Smads. In bacterial systems on the other hand, basically a single phosphate group is transferred, as in a relay race, from the cell surface receptor to the DNA. Bacterial systems also typically involve a smaller number of signaling proteins, i.e. their system size is smaller [8], [9].

Two component systems are found in nearly every bacteria and control myriad processes from nutrient sensing, chemotaxis, osmolarity control, quorum sensing and many others [10]–[12]. Most bacteria have many two component systems, and some are reported to have hundreds of them. Both the HK and the RR are paralogous gene families and they share significant amino acid and structural similarity within themselves [12]. It is possible therefore to imagine making use of cross-talk between pathways with similar structures to integrate signals into the final cellular decision. However despite a lot of research trying to look for examples of such cross-regulation, very few have been found [12]. The biochemical basis for cross-talk in vivo does exist with overexpression studies demonstrating that phosphotransfer between a HK and its noncognate RR is possible *in vivo*. However bacteria appear to use many methods to explicitly suppress cross-talk between two component systems. The known mechanisms of cross-talk suppression include: (i) bifunctional histidine kinases that act as a phosphatase for response regulators (ii) competition by the cognate RR that phosphotransfers with greater efficiency due to biochemical specificity and (iii) relatively low concentration of the HK to optimize the competition by the cognate RR [12].

There are also a few examples of situations where more than one HK signals through the same RR. For example, in the sporulation pathway of *B. subtilis*, four HK's can signal through a single response regulator, Spo0F [13]. Similarly, in the quorum sensing pathway of *V. harveyi*, three histidine kinases, LuxN, LuxQ and CqsS can phosphotransfer with the response regulator LuxU [14], [15]. These many-to-one branched pathways beg the question as to how bacteria can distinguish between signals originating from different HK's. The *V. harveyi* quorum sensing signal was studied in Ref. [15] which concluded that the bacteria could not distinguish between signals originating from the different HKs based on steady state values of a single phosphorylated RR alone. However the effects of cross-talk on the ability to distinguish between signals originating from different two-component systems have not yet been studied. This question gains significance given that bacteria appear to minimize cross-talk and do not appear to make use of it for cross-regulation [12].

To gain some insight into these issues, we turned to information theory. Information theory was developed in the late 1940s to ask abstract questions about general communication channels and has been used to gain insights about biological communication in the cell [16]–[18]. Information theory can be regarded as an application of probability theory to the problems of determining limits of information transmission in any communication channel, and it allows us to quantify the *quantity* of information that a network carries.

## Methods

From the point of view of information theory, a signal transduction network that takes an extra-cellular signal and converts it into a concentration of a transcription factor is a noisy communication channel whose task is to convey information about the extra-cellular signals to the decoding and the decision making apparatus in the nucleus of the cell [17], [19]. If the distribution of the extra-cellular signal is given by a joint probability distribution function 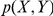, where 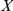 and 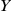 are the levels of the extra-cellular signals, the total uncertainty of 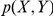 is measured by the Shannon entropy of their joint probability distribution function,
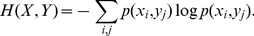
(1)


Here we follow the convention that the random variable is denoted by the capital letter, as in 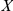, while the specific values it takes is the respective lower case letter, such as 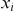. The information about the value of 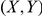 on the surface is encoded into the concentration of the output 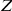, which in our case is the concentration of an activated transcription factor. This is decoded by the genetic architecture and the appropriate response determined. We assume here that the cell has developed optimal decoding methods over millions of years of evolution and concentrate only on the information present in the output signal, 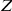. The information contained in 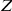 about the value of 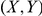 can be thought of as the reduction in uncertainty about 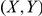 by knowledge of 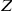. This is measured by a quantity called the *mutual information* between 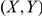 and 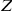 [20], denoted 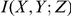, which is given by,
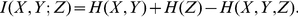
(2)


Now we can ask to what extent the cell can discriminate between the signals it receives from the two external ligands. Following Ref. [15], this is equivalent to asking how much the uncertainty in 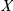 is decreased by knowledge of 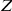, independent of the value of 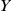, and can be estimated by the mutual information between 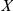 and 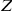 independently of 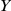, denoted 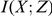. A similar calculation can be performed for 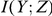.

The mutual information is usually calculated using logarithms to base 2 and measured in bits. One bit corresponds to knowledge about the state of a 2-state system. The information content is therefore an absolute measure and can be given a physical meaning. In general, if the mutual information between 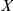 and 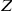 is 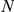 bits, the cell should be able to distinguish upto 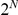 distinct states of 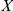 from knowledge of 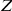, under the assumption of efficient decoding.

The physiological probability distribution function for the external input, the 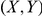 vector, is unknown. However since we are exploring the information processing capabilities of the networks in question, we can construct an arbitrary probability distribution function of the inputs. The simplest assumption is to start with a discrete distribution of 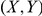 with equal probabilities, i.e. a discrete uniform distribution over a two-dimensional range. In physiological conditions it is certainly likely that the cell needs to distinguish between coarsely positioned discrete values or ranges of the external ligands than very small differences (though the latter may be appropriate for some sensory cells), hence we chose a 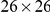 grid of 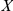 and 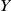 values spaced by 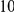 molecules from 0 to 250. The probability of seeing any of the combinations of 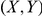 is therefore,
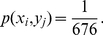
(3)


We keep this number fixed throughout this paper. This also sets the total uncertainty in the external distribution to be 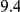 bits. We use this number to calculate the efficiency of information transfer later in the paper. Note that this exercise is equivalent to performing an experiment where the cell is exposed to each of the 676 different combinations of the external ligands many times, and a histogram of responses constructed. Thus assuming a uniform distribution of the external ligands is the most appropriate assumption from the point of view of an *in vitro* experiment on the lines of Ref. [21].

In terms of probability distribution functions of the output and the input, the mutual information can be written as,
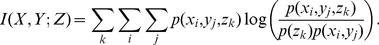
(4)

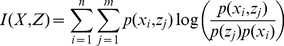
(5)where the joint probability distributions are defined in the usual way as,



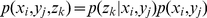
(6)

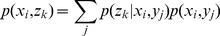
(7)


**Figure 1:**
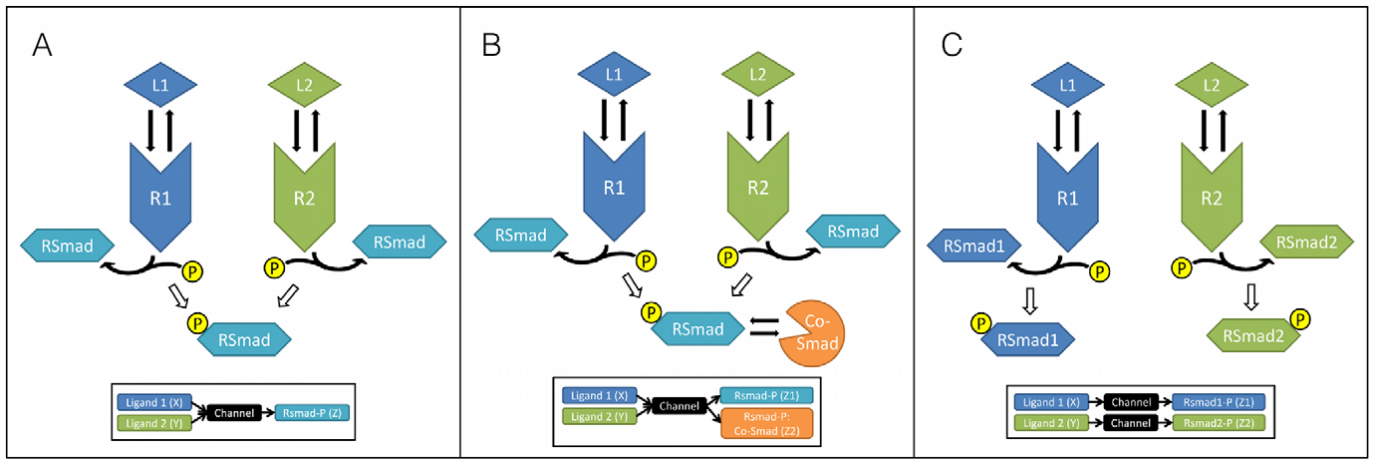
Smad signaling pathway network models. (**Model A**) A single channel of one RSmad with a single output. (**Model B**) A single channel with two outputs, the phosphorylated RSmad and the RSmad:Co-Smad heterodimer. (**Model C**) The dual channel with two distinct RSmads and two outputs. The insets diagram the information transmission topology of signal (ligand), channel, and output (complexes). Note that this diagram represents a phosphorylation reaction by the receptors, not a phosphotransfer.

**Figure 2:**
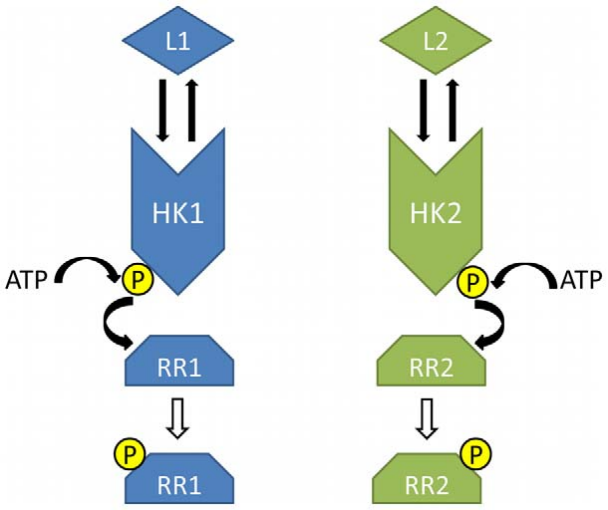
Bacterial two component system schematic model. Note that unlike the mammalian system a single phosphate group is transferred from the cell surface histidine kinase receptor to the response regulator.

To estimate these probabilities, we perform stochastic simulations of the signal transduction network using the Gillespie algorithm. For each one of the possible 676 inputs we carry out 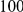 stochastic simulations of each network we consider using the Gillespie algorithm [22]. The Gillespie algorithm is an exact Monte Carlo simulation of the chemical Master equation that governs the stochastic evolution of the system. The models that we study are shown in Fig. 1 and are described as follows: (i) Fig. 1A shows the simplest model where two ligands operating through two surface receptors phosphorylate a single Smad. The output signal is the maximum accumulation of phosphorylated Smad. (ii) Fig. 1B shows the case where a protein called a Co-Smad binds to the activated Smad molecule. The signal at the nucleus then consists of a phosphorylated Smad and a heterodimer of a Smad with a Co-Smad, i.e. the output is bivariate. (iii) Fig. 1C shows the model with two Smads that are specific to the different receptors, and transduce the information to the nucleus. The output signal in this case are the maximum accumulations of the two phosphorylated Smads. (iv) Finally, Fig. 2 shows the network diagram of two bacterial two-component signaling systems. Here the receptor molecule, usually a histidine kinase, autophosphorylates on ligand binding, and the phosphate group is transferred to another protein called a response regulator. The output signal at the nucleus are the levels of the activated response regulator. The development of the Smad models is detailed in Table S1, Table S2 and Text S1. Development of the two-component model is detailed in Table S3 and Table S4.

The parameters for the simulations are mainly taken from previously published work on Smad signaling and bacterial signaling and are listed and discussed in Table S2 and Table S4. The stochastic simulations allow us to construct a distribution of output concentrations by binning at specified times. Previous work on Smad signaling indicates that it is not the temporal pattern of Smad accumulation but the accumulation in the nucleus that is the relevant physiological concentration [23], [24]. For the simple Smad model of Fig. 1A we therefore choose the maximum accumulation of the activated proteins as the output variable, or the 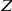 variable, and calculate the mean and the standard deviation of the maximum accumulation from the stochastic simulations. We then assume then that 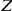 is normally distributed with the same mean and standard deviation. This is justified since the distribution of 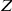 is the distribution of the mean of some other distribution, probably related to the extreme value distributions, and therefore by the Central Limit Theorem should be approximately normal.

The relevant distribution for each input combination is then binned to transform the normal distribution into a discrete distribution of 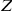-values. Since the information transfer naturally depends upon the bin size chosen to discretize 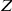, we decided to choose a bin size of 1. This is because due to discreteness of molecules this is the smallest relevant bin size. In effect we are assuming that the nucleus can make out differences of even one molecule of 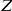, which is undoubtedly an overestimate of cellular information processing quantities. Thus the values of the mutual information we calculate should be considered as upper bounds of the information transfer with uniformly distributed inputs. After the binning the conditional probability distribution of 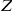 becomes:
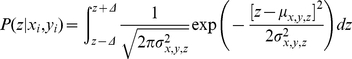
(8)where 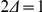 is the bin size. The values of the other probabilities required can be obtained from this equation by standard means using Eq. (7). These probabilities are then inserted into Eq. (4) and Eq. (5) to calculate the total and partial mutual information.

Note that the parameters are chosen so that the signal saturates above 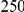 molecules of each ligand, as shown in Fig. S1. Therefore the ligand concentration covers the dynamic range of the signaling network.

Incorporation of the Co-Smad as in Fig. 1B converts the output from a scalar into a vector, 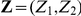, where 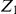 is the level of the activated Smad and 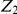 the level of the heterodimer. The probability distribution function of the output vector is therefore the joint probability distribution function of 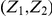. In accordance with our previous assumption we assume that this is given by the appropriately binned bivariate normal distribution, i.e.
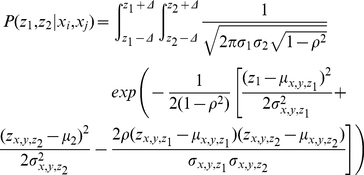
(9)where 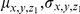 and 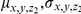 are the means and standard deviations of 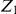 and 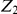, 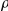 is the correlation coefficient and 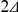 is the bin size. Here we take the bin size to be 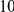 molecules to decrease the number of summations to be performed. As before the mean, standard deviation and correlation coefficients of the output is measured from the stochastic simulations. The mutual information between 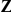 and the inputs 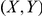 jointly or singly is given by Eq. (4) and Eq. (5) as before with the joint probability distribution function of 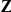 used in place of the univariate probability distribution function.

When we have two Smad proteins as in the model of Fig. 1C, we assume that the nucleus is only reading the levels of the phosphorylated RSmad and ignore dimerization, since our results show, as discussed later, that binding by the Co-Smad and dimerization or oligomerization are not likely to affect the information transfer. The input as before is the matrix of values 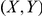 and the output now is the level of two phosphorylated Smad proteins, 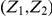. We again perform stochastic simulations to determine the mean and standard deviation and the correlation matrix of 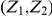 and use those values and the bivariate normal distribution Eq. (9) above to calculate the probabilities of 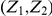 lying in discrete bins. These probabilities are then used to calculate the mutual information between the input signal and the output signal.

Bacterial two-component systems were modeled following Ref. [8], and the parameter values were mostly taken from the same reference. The system is schematically shown in Fig. 2, the reactions and parameter values are detailed in Table S3 and Table S4 and the dynamic range of the signal at these parameter values is shown in Fig. S2. As before stochastic simulations of the reactions were carried out to determine the mean and the standard deviation of the signal, which is here taken to be the steady state value of the phosphorylated response regulator. The signal itself is assumed to be distributed according to a bivariate normal distribution as above (Eq. 9), and the information measures are calculated as before. All the calculations performed for the case when the output was bivariate used the same bin-size 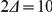.

## Results

### Two ligands and a single Smad

We begin from the simplest possible model of Smad signaling shown in Fig. 1A. In this model, two extracellular ligands can bind to their cognate receptor heterodimer. The bound complex can then recruit and phosphorylate Smad proteins which then become transcription factors. We assume that each BMP ligand does not interact with the other receptor pair; in other words, there is perfect specificity at the receptor level, but both receptors signal through a single phosphorylated Smad protein. The detailed reactions and the parameter values chosen are shown in Table S1 and Table S2. We ignore the role of the Co-Smad and oligomerization of the Smads initially (see below).

**Figure 3:**
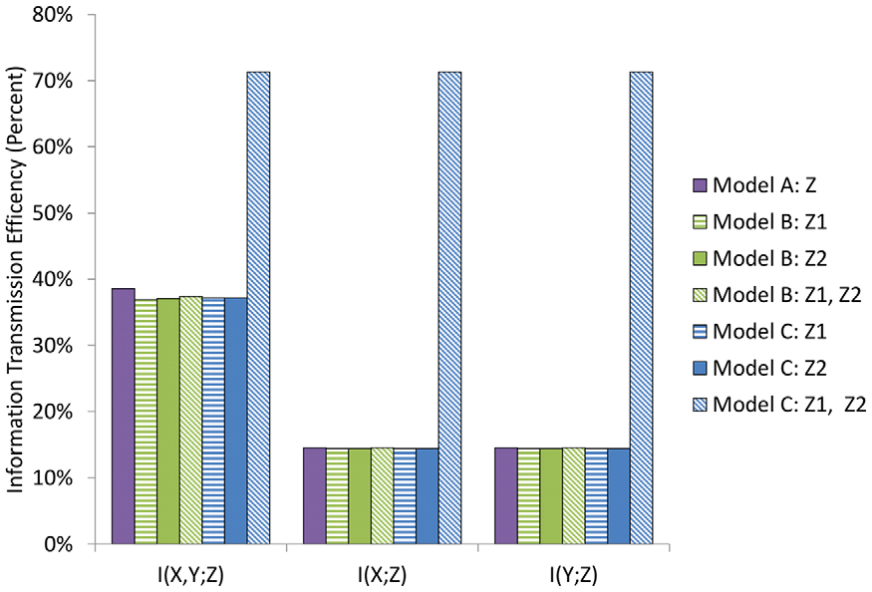
Summary of calculations of 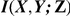, 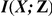 and 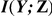 for the three Smad pathway network topologies considered in this paper. Information transfer efficiency as a percentage of the total uncertainly in the external distribution of ligands is shown in the 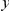-axis. Model A is the model with a single Smad (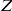), Model B is the model with a RSmad (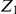) and a RSmad:Co-Smad heterodimer (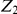) while Model C refers to the model with two RSmad proteins (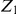 and 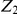). Information was calculated using either an individual output or both outputs (as is represented by Z1, Z2).

The results of our calculations are shown in Fig. 3 and in Table 1. We find that this simple network, which we call Model A, is not efficient in information transfer from the external ligand concentration vector 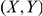 to the input 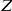. In fact as we show in Table 1, only about 3.6 bits of information about the vector 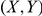 are contained in 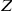, which corresponds to the ability to distinguish between only about 12 states of concentration values of the external ligand. This corresponds to about 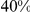 information transfer efficiency about the external distribution of 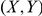.

**Table 1:** Mutual Information Values.

Model	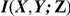	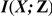
Model A	3.63	0.68
Model B	3.57	0.68
Model C	6.7	3.35

The mutual information is shown for each model at basal parameter values. Model A has only one phosphorylated protein, i.e. a single Smad as an output. Model B's output consists of the phosphorylated Smad as well as a Smad:Co-Smad dimer, and Model C's output consists of two different Smad proteins.

**Figure 4:**
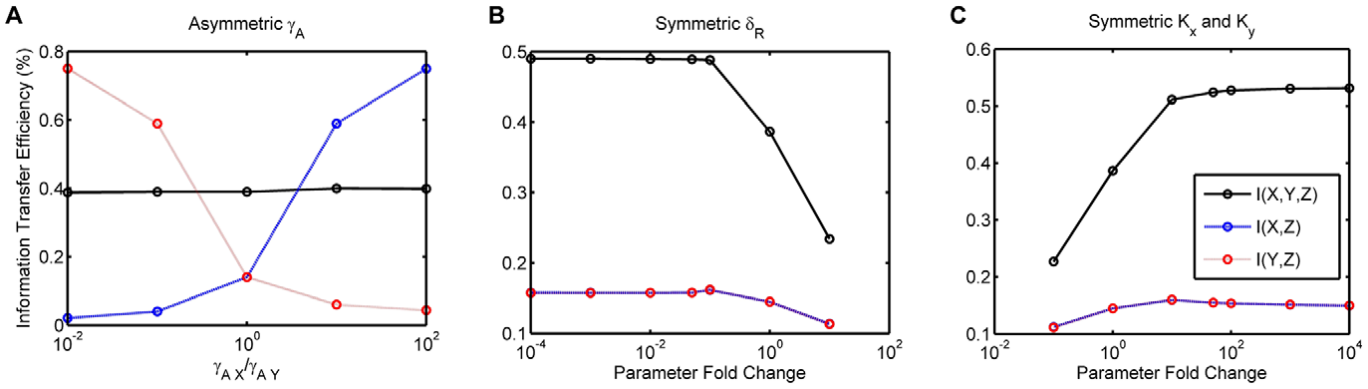
Parameter effects on information transmission for Model A. (**A**) Information transfer efficiency (as a percentage of the total uncertainly in the external distribution of ligands) vs. the ratio of the rates of association of the RSmad to the X and Y receptors. (**B**) Information transfer efficiency vs. symmetrical increase/decrease of receptor degradation rate from the standard parameter rate. (**C**) Information transfer efficiency vs. symmetrical increase/decrease of ligand binding rate from the standard parameter rate.

However the ability of the cell to discriminate between 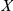 signals and 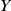 signals is even poorer. At our basal parameter values we find that about 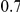 bits of information about 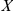 or 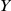 alone is contained in the level of 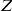, which implies that the cell cannot even tell if 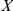 is high or low, since that requires one bit of information. This result is expected since both 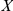 and 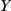 are activated in a completely symmetric way, so it is to be expected that the cell cannot distinguish between different levels of 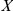 when the effects of 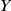 are potentially confounding. A possible way out for the cell to distinguish between ligands would be to increase the asymmetry in the kinetic responses elicited by the two ligands by, for example, making the phosphorylation rate of the RSmad by the receptor for 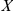 much higher than the other receptor. As shown in Fig. 4A we find that while this does lead to small increases in the information transmitted about 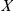, it is at the cost of information about 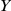. Thus, maximizing information transfer about both stimuli is only possible when all rates are symmetric, i.e. at the cost of the ability to discriminate. This is the same as in two-component signal transduction systems in bacteria [15].

Mutual information turned out to be rather insensitive to the parameter values that we choose for the simulation, as shown in Fig. 4B and C. Our parameter sensitivity analysis (Text S1, Fig. S3, Fig. S4, Table S5, Table S6) shows that most parameters had marginal effects on information transfer. The few parameters that could affect information transfer significantly are shown in Fig. 4. If the rate of receptor degradation is increased, it can decrease information transfer significantly, since the receptors degrade before a steady state binding equilibrium between the ligand and the receptors have been reached. However slowing the rate of degradation does not significantly increase information transfer, which plateaus at about 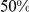 efficiency. Similarly, decreasing the equilibrium constant of binding between the ligand and the receptor leads to a decline in information transfer. However increasing the equilibrium constant beyond a point has no effect as information transfer again appears to plateau again at around 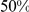. Note that the rate of increase of information transfer is at best logarithmic.

What determines where the curve plateaus? The cell cannot really distinguish accurately between a signal from 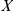 and a signal from 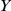. The level of 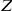 depends in fact on 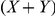 since both ligands feed into the signaling machinery that determines the level of 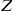. Therefore the best that the cell, or any decoding algorithm can do is to distinguish between different levels of 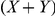. The curve plateau is therefore related to the best possible discrimination between different levels of 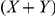 that is possible at the parameter values of the simulation.

### The Co-Smad does not increase information transfer

We then addressed the possible role of the Co-Smad in this network. In the biological network, the phosphorylated RSmad binds to a Smad protein called a Co-Smad and the heterodimer translocates to the nucleus and acts as a transcription factor. We wondered if the Co-Smad could help in translating small differences in the rate of phosphorylation of the RSmad by the two receptors into larger differences in the nucleus.

When we incorporate the Co-Smad (denoted as Model B), the output variable 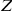 becomes a vector, 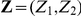, where 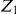 is the level of phosphorylated Rsmad and 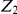 is the level of Rsmad:Co-Smad heterodimers. A diagram of this model is shown in Fig. 1B. Our calculations, summarized in Fig. 3 and Table 1, show that the coSmad heterodimer in fact does not contribute to the information transfer in the signaling network. This is not completely obvious since it could be imagined that at a given level of efficiency, adding 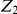 should increase total information transfer. As the data shows, efficiency at our basal parameter values is quite low, indicating that significant improvement is possible. However this cannot be achieved by adding a coSmad. The full details of this model can be found in Table S7.

Note that by the information processing inequality [20], information processing at an intermediate step in a Markov chain cannot increase the mutual information between the first step in the chain and the last. Therefore this inequality would predict that adding a Co-Smad should not be able to increase 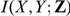. However adding a Co-Smad cannot increase 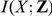 either since it acts symmetrically with respect to both channels since they transduce through a single Smad. Note that this implies that multimerization of the Co-Smad:RSmad complex cannot increase information transfer or signal discrimination either.

### Multiple Smad pathways

We now ask what the effect would be if we had two Smad proteins instead of one. In other words, if each ligand had, along with a preferred receptor, a preferred Smad protein. A diagram of the model is shown in Fig. 1C, and the reactions are detailed in Table S1 and the parameter values in Table S2. We refer to this model as Model C. We assume as before that each ligand binds only to its cognate receptor. However now each receptor has a preferred Smad that it phosphorylates, which we assume is identical with the rate for the case of the single Smad. The catalytic rate by which each receptor phosphorylates its noncognate Smad protein can be varied. We call this rate the level of cross-talk between the two pathways.

When the level of cross-talk is zero, each ligand has its own dedicated Smad protein. Therefore, as expected, the total information transferred approximately doubles, at base parameter values, to about 6.7 bits (see Fig. 3 and Table 1). That is equivalent to the capacity to distinguish between about 104 states of the input signal 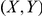, which is quite a large number of states. The absolute efficiency of information transfer at basal parameter values has now reached a respectable value, and is about 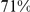. These results indicate that it is quite possible for signaling transduction networks to respond to relatively small changes in the levels of external ligands, and distinguish between many different states of these ligands merely by increasing the number of output proteins.

The ability to discriminate is as before measured by the mutual information between the output 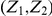 and the input signal 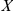 (or 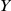) by summing up over all 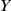 (or 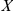). As shown in Fig. 3, we find a significant increase in the ability to discriminate with the mutual information 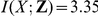 bits at basal parameter values, which corresponds to about 10 states of the ligand concentration 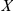. By using two Smads the cell has also restored the symmetry between the cell's ability to distinguish different levels of 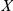 and different levels of 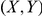 since the latter is approximately corresponds to 10 values of 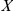 and 10 values of 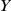, i.e. a total of 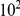 levels of 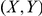.

### Cross-talk between Smad pathways

The above results are based on our calculations when the level of cross-talk is zero, i.e. each receptor talks with only its own cognate Smad. However most biological signaling pathways with multiple proteins usually have some cross-talk between proteins. Cross-talk between different proteins can be expected to decrease the efficiency of the information transmitted. To test what happens when the level of cross-talk increases, we then let each receptor phosphorylate the noncognate Smad at a fraction of the rate at which it phosphorylates its cognate Smad. This is implemented by changing the on-rate of binding of the non-cognate Smad with its non-cognate receptor from zero to some positive value, while the catalytic phosphorylation rate remains the same for all the cognate and non-cognate pairs. The ratio between the binding on-rate of the non-cognate pair with that of the cognate pair is thus a measure of the level of cross-talk, which can be varied both symmetrically, i.e. each receptor has the same amount of cross-talk as the other, or asymmetrically.

**Figure 5:**
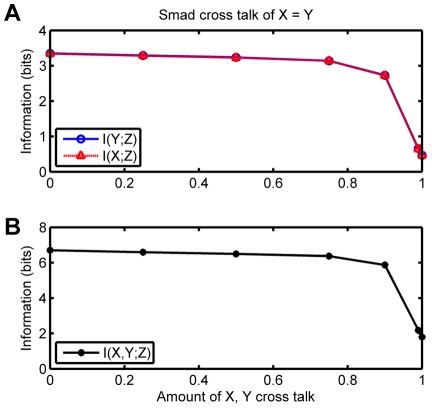
Information in bits vs fraction of cross-talk for the Smad Model C, with equal cross talk. Note that cross-talk is defined as the ratio of the on-rate of a Smad protein for the non-cognate receptor to the on-rate for its cognate receptor. When cross-talk is zero, only the cognate receptor can phosphorylate the Smad; when cross-talk is one, both receptors are equally efficient in phosphorylation of that Smad. In this plot the cross-talk between the receptor for X and output 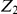 is the same as that between the receptor for Y with output 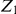. (**A**) Partial mutual information. (**B**) Total mutual information.

We find surprisingly that a significant level of cross-talk is tolerated before the information transmission efficiency decreases. As we show in Fig. 5, even when the effective phosphorylation of each receptor with the non-cognate Smad is 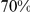 what it is with its cognate Smad, the total mutual information 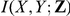 as well as the partial mutual information 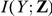 only marginally decreases compared to the case with no cross-talk. A significant decrease in the capacity of the channel requires that the cross-talk is greater than 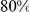. When the cross-talk is 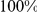, then as expected, both the Smad proteins are effectively the same, and the cell cannot do better than with a single channel. We find in fact that for total mutual information, it does a little worse, probably due to interference between the two pathways.

**Figure 6:**
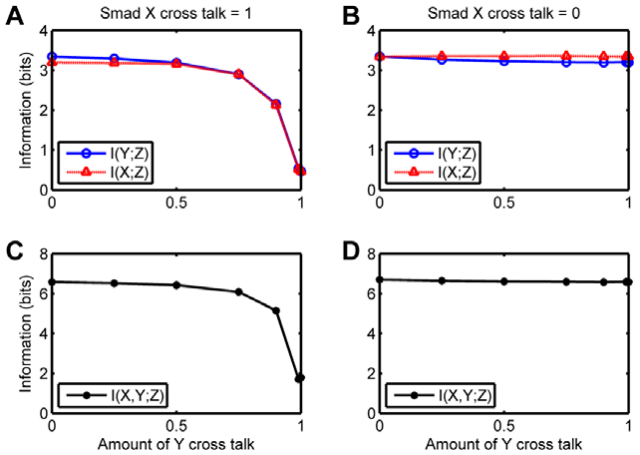
Mutual information as a function of cross-talk for Smad Model C when the cross-talk is varied asymmetrically. (**A** and **C**) Cross talk for the receptor for X with its non-cognate Smad held at 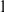. (**B** and **D**) Cross talk for the receptor for X with its non-cognate Smad held at 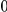. (**A** and **B**) Partial mutual information. (**C** and **D**) Total mutual information.

In Fig. 6 we show what happens when the cross-talk between one receptor-non-cognate Smad pair is kept fixed at either zero or one while the other varies. Here we see that if one receptor does not talk at all to its non-cognate Smad, it does not matter even if the cross-talk of the other receptor for the non-cognate Smad is 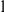; the mutual information is completely unaffected. If on the other hand the cross-talk between one receptor-non-cognate Smad pair is kept at 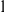, increasing the cross-talk of the other pair up from zero begins to adversely affect the information content of the channel only when the cross-talk crosses about 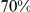. Therefore information content suffers only when the cross-talk efficiencies are symmetrically high.

This scenario has some interesting implications for protein evolution and information transfer. Due to this relation between cross-talk and 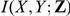 for both the symmetrical and the asymmetrical cases discussed above, there does not appear to be a strong tendency for minimization of cross-talk on the basis of information transfer alone. However if signaling is relatively robust against high levels of cross-talk, it is robust against having overlapping or partially redundant pathways. Redundancy has many protective advantages in biology, and in mammalian signaling for example, partially redundant pathways can compensate for defects in other pathways [25]. It also becomes possible to imagine the development of new functionalities from small mutations in signaling proteins as well as the development of cross-regulation wherein cross-talk is exploited to integrate signals coming from many external stimuli.

### Cross-talk in bacterial two component systems

We now turn to bacterial two component systems. The basic structure of a bacterial two component system is shown in Fig. 2. This consists of a cell surface receptor, usually a histidine kinase (HK) that can autophosphorylate when bound with its cognate ligand. The phosphate group can then be transferred to another protein molecule, generically called a response regulator (RR). The phosphorylated RR then turns on specific genes in the bacterial DNA [10], [11].

The main differences between the mammalian system and the bacterial system are the system size and the difference in the method of enzymatic activity i.e. the receptor molecules in bacteria autophosphorylate followed by a phosphotransfer to the response regulator. Mammalian cells on the other hand have receptor molecules that phosphorylate the cognate signaling protein, transferring a phosphate group usually present in excess in solution to the protein in question. It turns out that these differences do not necessarily lead to a change in total information transfer in the absence of cross-talk. Our calculations based on parameter values from [8] and ligand concentrations that almost cover the dynamic range of the system response show that two separate response regulators can transduce, in the absence of cross-talk, about 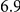 bits of information when taken together, which is about the same as the Smad system. The bacterial system size with these parameter values is about an order of magnitude smaller than the Smad system size as shown in Fig. S2. This is consistent with measured protein concentrations in many two component systems [8], [9].

**Figure 7:**
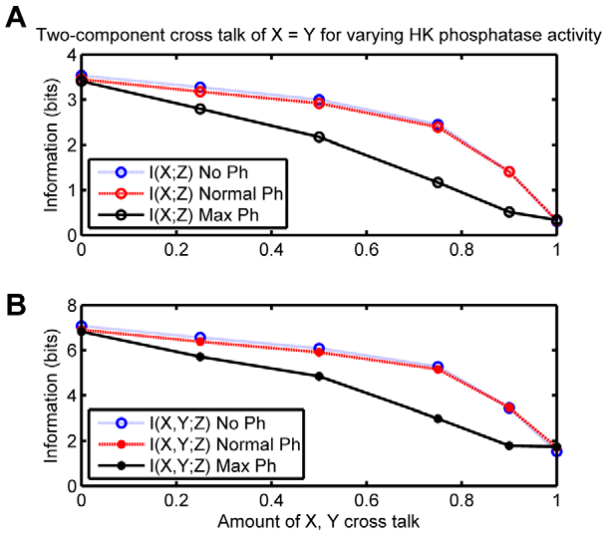
Information in bits vs. cross-talk for the two-component model, with equal cross talk and various HK phosphatase activities. (**A**) Partial mutual information and (**B**) total mutual information. The red line shows the default phosphatase activity where strength of phosphatase activity toward the non-cognate RR varies with level of cross talk. The blue line shows a system where the HK has no phosphatase activity. The black line shows where the strength of phosphatase activity toward the non-cognate RR is maximum regardless of the level of cross-talk.

**Figure 8:**
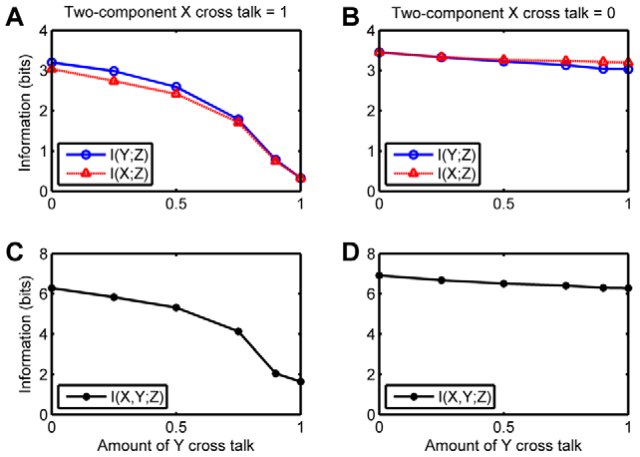
Information in bits vs. cross-talk for the two-component model, with asymmetric cross talk. (**A** and **C**) Cross talk for the receptor for X with its non-cognate Smad held at 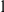. (**B** and **D**) Cross talk for the receptor for X with its non-cognate Smad held at 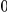. (**A** and **B**) Partial mutual information. (**C** and **D**) Total mutual information.

However when cross-talk is added to the system it shows a very different behavior. The mutual information 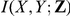, between the external ligands 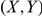 and the level of phosphorylated response regulators 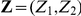 begins declining monotonically as cross-talk between the two HK's is symmetrically increased from zero as shown in Fig. 7. The mutual information between one external ligand and the output 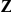 also declines in a similar manner. This is in sharp contrast with the behavior of the mammalian system as discussed above. The bacterial cell is more robust to cross-talk when it is only one-sided, i.e. only one HK can phosphotransfer to both RRs. In this case, as we see in Fig. 8A the decline in 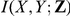 and the decline in 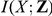 is much slower. However if one HK is already promiscuous then increasing the cross-talk of the other leads to an even sharper decline in both total mutual information as well as partial mutual information (Fig. 8B).

Modeling studies have argued that phosphatase activity of a HK with respect to its RR can buffer the system against cross-talk by dephosphorylation of weak signals from a non-cognate HK. However this method is probably unlikely to be very efficient when both external ligands are present and therefore both HKs are being activated. We tested this by simulating the system when the phosphatase activity of the HK was kept at a maximum regardless of cross-talk (black line), when the phosphatase activity for the non-cognate RR varies proportionately with level of cross-talk (red line), and when the HKs have no phosphatase activity (blue line) Fig. 7. As shown in Fig. 7 the phosphatase activity of the HK has no impact on cross-talk when both external ligands are present and the mutual information measures 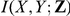 and 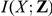 decrease monotonically. In the case of maximum phosphatase activity a sharper decline is seen, which may be a consequence of suppression of the signal to the cognate RR due to the high phosphatase activity.

**Figure 9:**
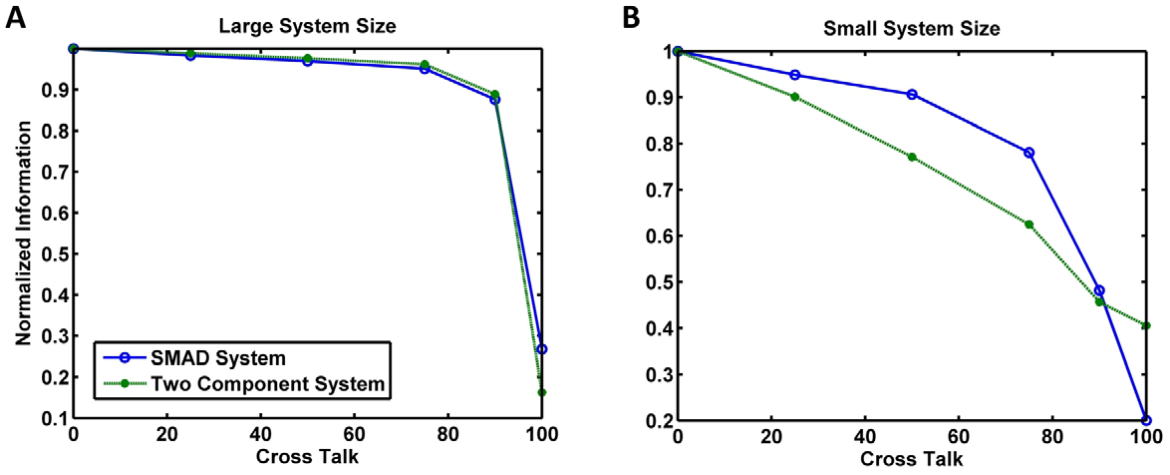
Information in bits vs. symmetrically varying cross-talk, a comparison of the effects of system size and the mode of phosphotransfer. (A) The Smad system and the two-component system for large system sizes (B) the Smad systems and the two-component system for small system sizes.

In order to understand whether the degradation of information content was due to the difference in system size or due to the kinetic differences between the two pathways, we took the two-component model and changed parameters (Table S8 and Fig. S9) until we obtained a dynamic range that was approximately equivalent to the Smad model. Similarly, we took the Smad signaling model and changed parameters to obtain a model that yielded protein numbers that were of the same order as that of the two-component model (Fig. S10). Results of the two large-protein-number models are are shown in Fig. 9A, and they indicate that in fact at high protein numbers the two modes of signal transduction are identical. The results from the two small-protein-number models, shown in Fig. 9B, suggest that at small protein numbers there is still some difference between the two cases, that could be due to the small remaining difference in protein numbers, the higher level of noise of the two-component circuit, or the mode of receptor action.

Why do the smaller system sizes that we simulate in this work show a greater degradation of information with increasing cross-talk? Smaller protein numbers are associated with larger relative fluctuations due to the intrinsic stochasticity of signaling networks. Symmetric crosstalk not only increases the total noise in the system, it also leads to decrease in the absolute number of useful molecules for each signaling channel, thereby further decreasing the signal to noise ratio of each channel. This could very well be the reason why we see increasing sensitivity to cross-talk with a smaller system size.

The monotonic decline in information transfer with increasing cross-talk seen in our calculations suggest that small signaling systems, such as those characteristic of some bacterial two-component networks, cannot function efficiently in the presence of cross-talk without increasing the number of signaling proteins by an order of magnitude or so. Energetically it is cheaper to use two independent signaling pathways for transducing information, as they can transfer as much information with a smaller number of proteins. This suggests that evolution should have led to two component systems evolving to be relatively insulated from each other, as cross-talk would lead to a decline in fitness. This could be one reason why we do not find much cross-regulation between different two-component pathways. We can also predict, based on these arguments, that if cross-talk is introduced between two two-component pathways (by say a lateral gene transfer event), we should initially see a decline in fitness, and evolution should eventually drive the system to eliminate cross-talk between these pathways altogether.

## Discussion

We have used information theoretic methods to study the transmission of information in simple signaling networks based on the Smad signaling pathway of the TGF-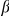 proteins in mammalian cells and two-component systems in bacteria. It is often assumed that what is important in gene circuits or the cell in general is bistability, i.e. having two states – ‘on’ and ‘off’. However in principle the information transmitted by a simple signaling pathway like the Smad signaling pathway can allow the cell to perform much more sophisticated information processing than simple binary decisions [26].

It is not clear whether signal transduction networks in cells actually transduce more than one bit of information. Recent experimental studies on Tumor Necrosis Factor Alpha signaling have claimed that only one bit of information is carried in several important signaling networks [21]. However in principle many signal transduction networks appear to have the ability to distinguish more than binary levels of extra-cellular signals. Some sensory cells like neurons and hair cells in the ears have extremely accurate sensing capabilities, that have been optimized over millions of years of evolution. It has therefore also been argued that evolution should have optimized the information transmission capabilities of signal transduction networks [17]. In this paper however we do not use the optimality assumption but rather ask whether the information transmitted to the nucleus could potentially allow the cell to reconstruct the distribution of the external signals that led to the signal. In particular we ask whether these signaling pathways have the capacity to allow the cell to distinguish between signals received by two external ligands.

Our results are based on calculations of two measures, the total mutual information 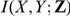 and the partial mutual information 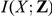. The total mutual information 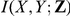 tells us the maximum number of states of the external ligand concentration 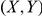 that the cell could in principle distinguish, assuming efficient decoding mechanisms exist. Similarly, 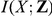 tells us the number of states of the ligand concentration 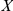 that the cell could distinguish from knowledge of 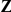 alone. Both of these measures depend upon parameter values and concentrations, as well as upon the topology of the network. In this study we assume reasonable parameters and calculate the information transmission measures at these parameter values. We then vary all parameters by large amounts to see whether the qualitative results are sensitive to the choice of parameter values. We find that our qualitative results are very robust against wide variations in most parameter values. Parameters adjusted are shown in Table S5, Table S6 and Table S7 and the results are shown in Fig. S5, Fig. S6, Fig. S7 and Fig. S8.

We find, in agreement with previous results [15] that the cell cannot distinguish between different levels of the external ligands 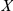 or 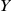 based on the level of phosphorylated Smad protein if the receptors for the two external ligands are symmetric in terms of their effective rates of phosphorylation of the Smads. While some specificity can be introduced by making the receptors asymmetric, this is at the cost of one of the two external signals. The ability to discriminate is not helped by addition of a Co-Smad to the system.

However we find that addition of another output protein, i.e. another R-Smad, increases both the total information carried as well as dramatically increases the cell's ability to distinguish between different levels of the external ligands. While in the case of a single Smad, the cell could not distinguish even between high and low levels of a single external ligand, with two Smads the cell can, in principle, distinguish between 10 different levels. The multiplicity of signaling proteins that carry information to the nucleus in pathways like the Smad signaling pathway are probably a direct consequence of this dramatic increase in information transmission.

It should be expected that as the cross-talk between the receptors of the two output proteins, 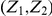 increases, it leads to a decrease in the ability of the cell to discriminate and in the total information carried by the channel. When cross-talk is 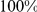 in both directions, effectively both 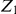 and 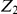 are indistinguishable from each other and we find, as expected, that no additional information is carried by the communication channel compared to a single 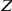 protein. However as the level of cross-talk is decreased below 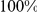, we find a relatively steep increase in both measures that almost reach a plateau by the time the cross-talk drops to below 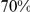. In other words we find that contrary to intuition, a high level of cross-talk is not very deleterious to information transmission by the Smad pathway, or other similar pathways, in mammalian cells.

This result has potentially significant implications. Consider the situation where a single signaling pathway is altered by a heterozygous mutation in one allele of the gene corresponding to a Smad-like protein. If the heterozygous mutation is in an important residue and it leads to one of these proteins becoming preferred for a previously existing function, or acquiring a new function, it would result in a significant increase in information transfer, possibly conferring an evolutionary advantage that could lead to the mutation being fixed in the population. For example sequence analysis shows that the human Smad proteins cluster into two groups, one associated with BMP signaling and the other associated with TGF-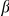 signaling [27] and both clusters share significant sequence similarity. It is possible therefore that each cluster arose by mutations in a single protein that was beneficial because of the resulting increase in information transfer despite the high level of cross-talk. Similarly BMP2 and BMP4 share 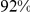 sequence similarity but play some non-redundant roles in cellular signaling. It is possible therefore that BMP 2 and 4 could have originated by mutations in a single BMP protein that created ‘new’ extra-cellular ligands with different receptor specificity and 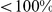 cross-talk with each other, leading to a significant increase in information transmission. Increases in information transmission due to such mutations could be one of the important sources of positive selection of mutations in signal transduction.

Another very common scenario is duplicate genes that are ubiquitous in human and other genomes. About 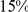 of the human genome consists of duplicate genes, many of which have diverged in function [28]. The creation of a duplicate gene would pave the way for gradual divergence of each gene [25]. The acquisition of new functions again would be crucially helped by the fact that the cell can deconstruct signals coming from each protein despite cross-talk. It is possible that signaling pathways depending upon closely related sets of genes diverged from each other due to such processes. As long as the cross-talk between these pathways is not close to one, there are no deleterious effects on the original pathway. Furthermore, the existence of overlapping pathways does provide protection due to development of redundancy in the cell, and leads to the possibility of cross-regulation, i.e. integration of multiple signals into the same decision process [25]. These results are not exclusive to the Smad pathway as there are a number of mammalian pathways with similar topology, such as the Jak-Stat pathway [6], where robustness against cross-talk when surface receptors are efficient kinases for transcription factor molecules may have played a role in the development of complexity in signaling networks.

The dominant cause of the relative insensitivity of the system towards increasing cross-talk appears to be the system size. In smaller systems such as bacterial two-component systems, we see an almost monotonic decline of total mutual information and partial mutual information when cross-talk exists between two HKs for their non-cognate RRs. The sensitivity to cross-talk in smaller two-component systems may be one reason why bacteria, who can have hundreds of such systems, expend considerable effort to avoid cross-talk and keep them insulated from each other. It is interesting to note that many researchers had assumed that two-component signaling should naturally allow for cross-regulation between different pathways; however despite significant efforts, few examples of cross-regulation have been found [12]. Cross-regulation is not possible between systems where interference between two pathways leads to attenuation of information transfer. Our calculations would therefore predict that if cross-talk were introduced in a bacterium due to either lateral gene transfer or artificially, evolution would again tend to minimize the cross-talk between these two systems in order to overcome the fitness loss due to aberrant information transfer. Of course some bacterial signaling systems also involve thousands of proteins and are therefore large in the sense implied in this paper. Our analysis would predict that these larger systems are more likely to be insensitive to cross-talk, or to exploit it, compared to the smaller two-component systems.

We have not studied the effect that different input distributions may have on cross-talk between related signaling pathways, though we believe that they are unlikely to change our qualitative results. This is in accordance with Mehta et. al. [15] who found that different distributions of the ligand did not affect their results for a single transcription factor. It is possible that the efficiency of information transfer increases when the distribution of the extra-cellular ligand is different from the uniform distribution. The uniform distribution also has the maximum amount of uncertainty. Our results however easily translate into an experiment where a cell is exposed to different concentrations of two extracellular ligands repeatedly and the levels of the activated transcription factor measured. The histogram of these levels for each input combination is precisely the conditional probability distribution, 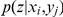.

The dependence of our results on system size may be because smaller systems have a higher noise to signal ratio due to the intrinsic stochasticity of chemical reactions. We are currently studying this relationship with the aim of uncovering a more precise quantitative relation between system size and the effect of cross-talk. Further work is also needed to understand how gene transcription networks can interpret signals coming from systems with an innately high level of cross-talk. Moreover, in future work we also need to understand what happens when there is cross-talk between more than two pathways at the same time. This is particularly relevant for TGF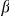 signaling and BMP signaling, since both of them have at least three Smad homolog's that are involved in information transfer from the receptor to the nucleus. Finally our analysis also leads to the design of experiments to be performed that can confirm or falsify our predictions and uncover how cells make sense of the world in the presence of cross-talk.

## Supporting Information

Figure S1 **Standard Output for Two Output R and RC.** The x- and y-axes correspond to the initial ligand amount of ligand X and Y respectively. The z-axis is the average maximum accumulation of the outputs: phosphorylated RSmad and the RSmad:Co-Smad heterodimer. Note that the outputs saturate at maximum initial ligand amounts.
(TIF)

Figure S2
**Standard Output for Phosphorylated Response Regulator.** The x- and y-axes correspond to the initial ligand amount of ligand X and Y respectively. The z-axis is the average maximum accumulation of the output: phosphorylated response regulator. Note that the outputs saturate at maximum initial ligand amounts. This was done with 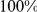 cross talk and shows response regulator 1.(TIF)

Figure S3
**Dynamic Range of Maximal R-Smad Accumulation for Bilateral Fold Changes of 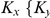 and 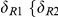.** This figure demonstrates the effect of symmetrically varying the rates of receptor degradation and ligand binding. Note that the panels increase/decrease symmetrically. The x- and y-axes correspond to the initial ligand amount of ligand X and Y respectively. The z-axis is the average maximum accumulation of the output, phosphorylated RSmad.(TIF)

Figure S4
**Dynamic Range of Maximal R-Smad Accumulation for Unilateral Fold Changes of 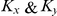 and 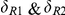.** This figure demonstrates the effect of asymmetrically varying the rates of receptor degradation and ligand binding. Note that the panels become skewed as the rates are increased/decreased asymmetrically for X and Y. The x- and y-axes correspond to the initial ligand amount of ligand X and Y respectively. The z-axis is the average maximum accumulation of the output, phosphorylated RSmad.(TIF)

Figure S5
**Effect of the Ligand On rate, 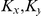 on information transfer.** The ligand on rate was increased or decreased 10-fold and 1000-fold and tested across a range of cross-talk values.(TIF)

Figure S6
**Effect of the Ligand Off rate, 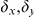 on information transfer.** The ligand off rate was increased or decreased 10-fold and 1000-fold and tested across a range of cross-talk values.(TIF)

Figure S7
**Effect of the Ligand On rate, 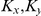 on information transfer.** The ligand on rate was increased or decreased 10-fold and 1000-fold and tested across a range of cross-talk values.(TIF)

Figure S8
**Effect of the Ligand Off rate, 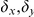 on information transfer.** The ligand off rate was increased or decreased 10-fold and 1000-fold and tested across a range of cross-talk values.(TIF)

Figure S9 **The Dynamic Range of the Large Two-Component Model.** Falls within the dynamic range of the small smad model.
(TIF)

Figure S10 **The Dynamic Range of the Small SMAD Model.** Is of the same order of magnitude as the two-component model.
(TIF)

Table S1 **Smad Model Reactions.**
(DOCX)

Table S2 **Parameters and Initial Amounts for Smad Model.**
(DOCX)

Table S3 **Two-Component Model Reactions.**
(DOCX)

Table S4 **Parameters and Initial Values for Two-Component Model.**
(DOCX)

Table S5 **Effect of Symmetric Parameter Change.**
(DOCX)

Table S6 **Effect of Asymmetric Parameter Change.**
(DOCX)

Table S7 **Model 1 Information and Entropy.**
(DOCX)

Table S8 **Parameters and Initial Values for Large Dynamic Range Two-Component Model.**
(DOCX)

Text S1 **Supplementary Information and Detailed Model Development.**
(DOCX)
