# Supplementary figures and images for: Cross-Talk and Information Transfer in Mammalian and Bacterial Signaling
Source: PLoS One. 2012 Apr 18;7(4):e34488. doi: 10.1371/journal.pone.0034488 (PMC3329486; doi:10.1371/journal.pone.0034488)

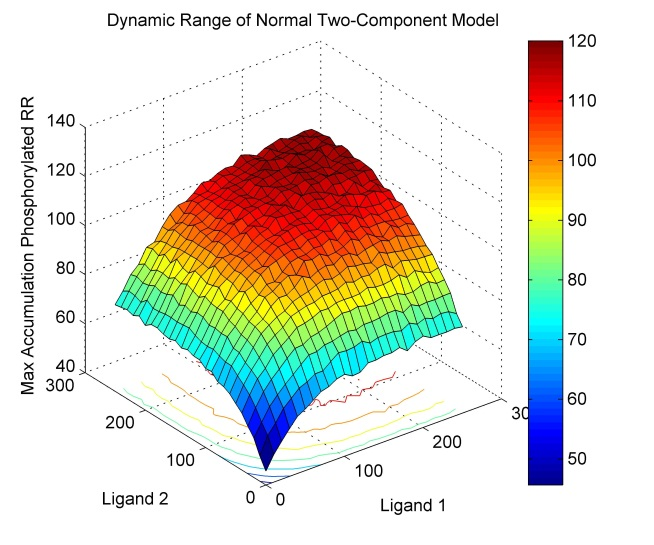

Supplement: Figure S2 — Standard Output for Phosphorylated Response Regulator. The x- and y-axes correspond to the initial ligand amount of ligand X and Y respectively. The z-axis is the average maximum accumulation of the output: phosphorylated response regulator. Note that the outputs saturate at maximum initial ligand amounts. This was done with cross talk and shows response regulator 1. (TIF) [file pone.0034488.s002.tif]

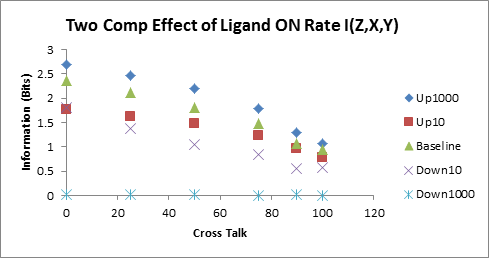

Supplement: Figure S5 — Effect of the Ligand On rate, on information transfer. The ligand on rate was increased or decreased 10-fold and 1000-fold and tested across a range of cross-talk values. (TIF) [file pone.0034488.s005.tif]

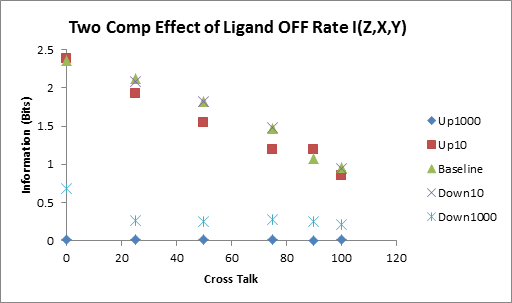

Supplement: Figure S6 — Effect of the Ligand Off rate, on information transfer. The ligand off rate was increased or decreased 10-fold and 1000-fold and tested across a range of cross-talk values. (TIF) [file pone.0034488.s006.tif]

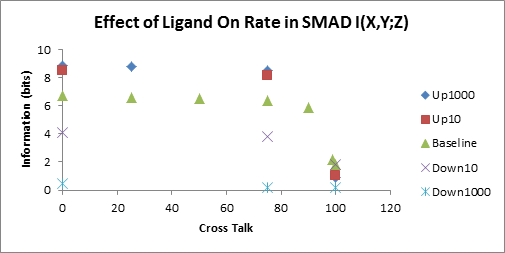

Supplement: Figure S7 — Effect of the Ligand On rate, on information transfer. The ligand on rate was increased or decreased 10-fold and 1000-fold and tested across a range of cross-talk values. (TIF) [file pone.0034488.s007.tif]

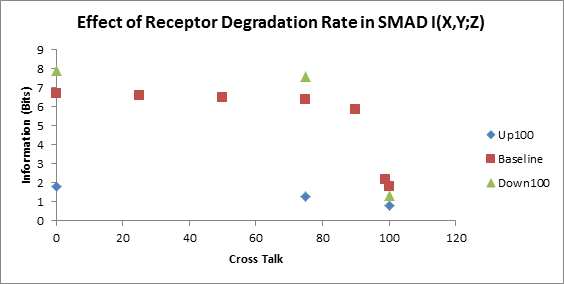

Supplement: Figure S8 — Effect of the Ligand Off rate, on information transfer. The ligand off rate was increased or decreased 10-fold and 1000-fold and tested across a range of cross-talk values. (TIF) [file pone.0034488.s008.tif]

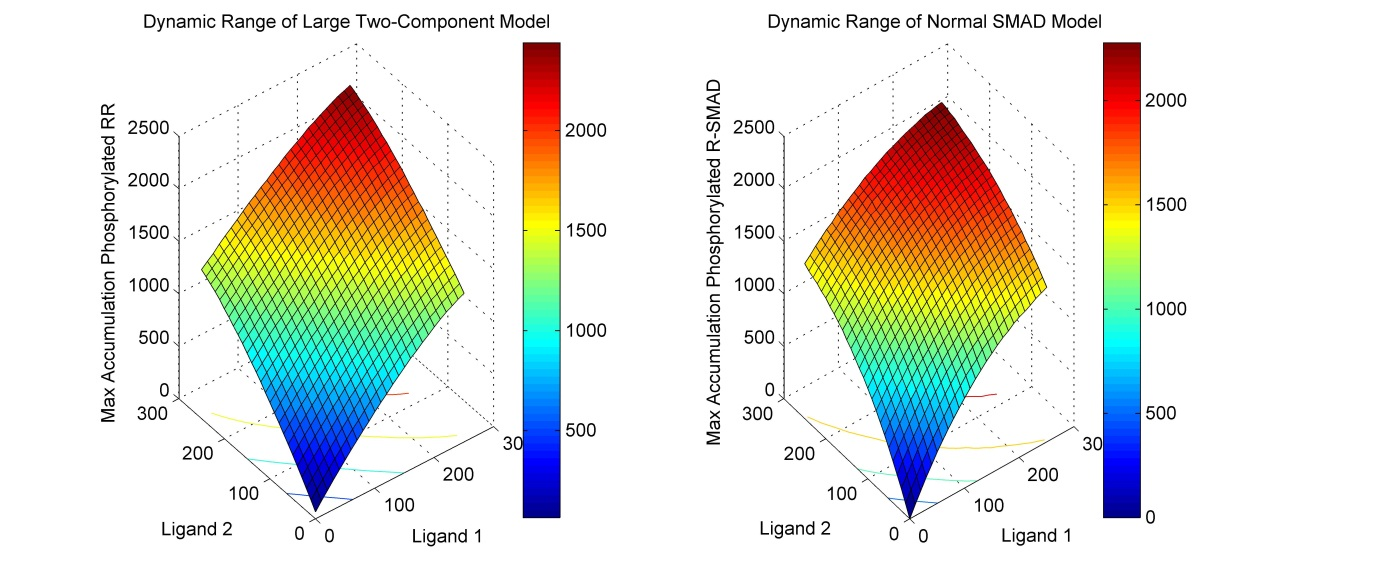

Supplement: Figure S9 — The Dynamic Range of the Large Two-Component Model. Falls within the dynamic range of the small smad model. (TIF) [file pone.0034488.s009.tif]

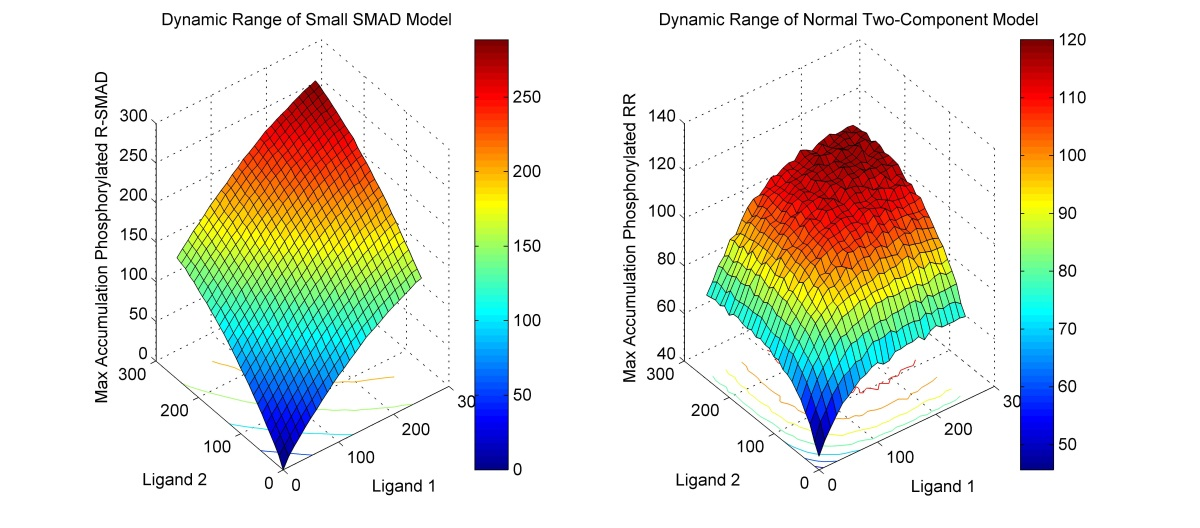

Supplement: Figure S10 — The Dynamic Range of the Small SMAD Model. Is of the same order of magnitude as the two-component model. (TIF) [file pone.0034488.s010.tif]
