# Supplementary material for: Cross-Talk and Information Transfer in Mammalian and Bacterial Signaling
Source: PLoS One. 2012 Apr 18;7(4):e34488. doi: 10.1371/journal.pone.0034488 (PMC3329486; doi:10.1371/journal.pone.0034488)
Supplement: Table S1 — Smad Model Reactions. (DOCX) [file pone.0034488.s011.docx]

Table S1. Smad Model Reactions

|  | **Reaction** | **Forward Rate** | **Reverse Rate** | **Description** |
| --- | --- | --- | --- | --- |
| 1 | R1+L1 <-> R1:L1 | k_X_ | δ_X_ | Ligand binds receptor |
| 2 | R2+L2 <-> R2:L2 | k_Y_ | δ_Y_ | Ligand binds receptor |
| 3 | R1 -> null | δ_R1_ |  | Receptor internalization/degredation |
| 4 | R2 -> null | δ_R2_ |  | Receptor internalization/degredation |
| 5^2^ | ri+Rj:Lj<->ri:Rj:Lj | γ_aij_ | γ_bij_ | rsmad binds receptor/ligand |
| 6^2^ | ri:Rj:Lj->Rj:Lj + ri:p | γ_cij_ |  | receptor phosphorylates and releases rsmad |
| 7 | r:p-> r | δ_P_ |  | rsmad-p dephosphorylation |
| 8 | r:p + C <-> r:p:C | μ | λ | rsmad-p binds cosmad |

Note: i = 1 or 2 for rsmad1 or rsmad2; j = 1 or 2 for Receptor/Ligand 1 or 2. All reactions were taken from (Nakabayashi & Sasaki, 2009) except where noted (2), and Michaelis-Menten kinetics were used for phosphorylation of an rsmad by a receptor:ligand complex.
