# Supplementary material for: Cross-Talk and Information Transfer in Mammalian and Bacterial Signaling
Source: PLoS One. 2012 Apr 18;7(4):e34488. doi: 10.1371/journal.pone.0034488 (PMC3329486; doi:10.1371/journal.pone.0034488)
Supplement: Table S2 — Parameters and Initial Amounts for Smad Model. (DOCX) [file pone.0034488.s012.docx]

Table S2. Parameters and Initial Amounts for Smad Model

| **Parameter** | **Standard Rate** | **Units** | **Description** |
| --- | --- | --- | --- |
| k_X_ = k_Y_^2^ | 1.0e-5 | 1/(molecule*second) | Ligand association rate |
| δ_X_ = δ_Y_^1^ | 5e-5 | 1/second | Ligand dissociation rate |
| δ_R1_ = δ_R2_^1^ | 5e-4 | 1/second | Receptor degredation rate |
| δ_P_^1^ | 6.6e-3 | 1/second | rsmad dephosphorylation rate |
| μ^1^ | 1.3e-6 | 1/(molecule*second) | rsmad:Cosmad association Rate |
| λ^1^ | 0.016 | 1/second | rsmad:Co dissociation rate |
| γ_a11_= γ_a22_^2^ | 4.0e-6 | 1/(molecule*second) | like R:L + rsmad association rate |
| γ_a12_= γ_a21_^2^ | 0 - 4.0e-6 (variable) | 1/(molecule*second) | unlike R:L + rsmad association rate |
| γ_b11_= γ_b22_ = γ_b12_= γ_b21_^2^ | 1.0e-4 | 1/second | R:L + rsmad dissociation rate |
| γ_c11_= γ_c22_= γ_c12_= γ_c21_^2^ | 2 | 1/second | Phosphotransfer/dissociation rate |
| L1, L2 | 0 – 260^1^ | molecule | Ligand 1, 2 |
| R1, R2 | 250^1^ | molecule | Receptor 1, 2 |
| r, r1, r2 | 10000 each^2^ | molecule | Rsmad 1, 2 |
| C | 10000^2^ | molecule | Co-Smad |
| r:p, r1:p, r2:p | 0^1^ | molecule | Phosphorylated Rsmad |

Note: Parameters and values were taken from 1. (Nakabayashi & Sasaki, 2009) or 2. based on appropriate ranges from similar rates from (Nakabayashi & Sasaki, 2009).
