# Supplementary material for: Cross-Talk and Information Transfer in Mammalian and Bacterial Signaling
Source: PLoS One. 2012 Apr 18;7(4):e34488. doi: 10.1371/journal.pone.0034488 (PMC3329486; doi:10.1371/journal.pone.0034488)
Supplement: Table S3 — Two-Component Model Reactions. (DOCX) [file pone.0034488.s013.docx]

Table S3. Two-Component Model Reactions

|  | **Reaction** | **Forward Rate** | **Reverse Rate** | **Description** |
| --- | --- | --- | --- | --- |
| 1 | HK1+L1 <-> HK1:L1 | k_X_ | δ_X_ | Ligand binds receptor |
| 2 | HK2+L2 <-> HK2:L2 | k_Y_ | δ_Y_ | Ligand binds receptor |
| 3 | HK -> HKp | K_ap_ |  | HK autophosphorylation |
| 4 | HK:L ->HKp:L | K_lp_ |  | HK autophosphorylation with ligand bound |
| 5 | HKp ->HK | K_ad_ |  | HK dephosphorylation regardless of ligand |
| 6 | HKi+RRj<->HKi:RRj | K_bij_ | k_d_ | HK binds RR regardless of phosphorylation state |
| 7 | HKp:RR ->HK:RRp | k_pt_ |  | HK phosphotransfer to RR |
| 8 | HK:RRp -> HK:RR | k_pd_ |  | HK phosphatase activity on RR |
| 9 | RRp -> RR | d_phos_ |  | RR dephosphorylates |
| 10 | L -> null | δ_L_ |  | Ligand degrades |
