# Supplementary material for: Cross-Talk and Information Transfer in Mammalian and Bacterial Signaling
Source: PLoS One. 2012 Apr 18;7(4):e34488. doi: 10.1371/journal.pone.0034488 (PMC3329486; doi:10.1371/journal.pone.0034488)
Supplement: Table S4 — Parameters and Initial Values for Two-Component Model. (DOCX) [file pone.0034488.s014.docx]

Table S4. Parameters and Initial Values for Two-Component Model

| **Parameter** | **Standard Rate** | **Units** | **Description** |
| --- | --- | --- | --- |
| k_X_ = k_Y_^4^ | 3.4E-4 | 1/(molecule*second) | ligand association rate |
| δ_X_ = δ_Y_^4^ | 0.5 | 1/second | ligand dissociation rate |
| k_ap_^3^ | 0.001 | 1/second | HK autophosphorylation |
| k_lp_^3^ | 0.1 | 1/second | HK autophosphorylation w/ L |
| k_ad_^3^ | 5E-4 | 1/second | HK dephosphorylation |
| kb_11_=kb_22_^3^ | 3.4E-4 | 1/(molecule*second) | cognate RR+HK binding |
| kb_12_= kb_21_^4^ | 0 -3.4E-4 (variable) | 1/(molecule*second) | non-cognate RR+HK binding |
| k_d_^3^ | 0.5 | 1/second | RR+HK unbinding |
| k_pt_^3^ | 1.5 | 1/second | phosphotransfer |
| k_pd_^3^ | 0.05 | 1/second | HK phosphatase |
| dphos^3^ | 6.6E-3 | 1/second | RRp dephosphorylation |
| δ_L_^4^ | 5E-5 | 1/second | ligand degradation |
| L1, L2^4^ | 0 – 250^3^ | molecule | ligand 1, 2 initial amount |
| HK1, HK2^3^ | 250^3^ | molecule | HK 1, 2 initial amount |
| RR1, RR2^3^ | 8824 each^3^ | molecule | RR 1, 2 initial amount |

Note: Initial Values and parameters were taken from 3. (Igoshin, Alves, & Savageau, 2008) or 4. based on appropriate ranges from similar rates from (Igoshin, Alves, & Savageau, 2008).
