# Supplementary material for: Cross-Talk and Information Transfer in Mammalian and Bacterial Signaling
Source: PLoS One. 2012 Apr 18;7(4):e34488. doi: 10.1371/journal.pone.0034488 (PMC3329486; doi:10.1371/journal.pone.0034488)
Supplement: Table S5 — Effect of Symmetric Parameter Change. (DOCX) [file pone.0034488.s015.docx]

Table S5. Effect of Symmetric Parameter Change

|  |  | Percent Change in Information (% of Bits) | | | Absolute Change in Efficiency (%) | | |
| --- | --- | --- | --- | --- | --- | --- | --- |
|  | Fold  Change | I(X,Y;Z) | I(X;Z) | I(Y;Z) | $\frac{I\left( X,Y;Z \right)}{H\left( X,Y;Z \right)}$ | $\frac{I\left( X;Z \right)}{H\left( X;Z \right)}$ | $\frac{I\left( Y;Z \right)}{H\left( Y;Z \right)}$ |
| δ_P_ | x10 | -3.43% | -1.21% | -1.09% | -1.28% | -0.20% | -0.18% |
|  | x0.1 | -5.18% | -1.98% | -2.37% | -1.97% | -0.31% | -0.37% |
| δ_R_ | x10 | -40.89% | -22.45% | -22.69% | -15.25% | -3.11% | -3.15% |
|  | x0.1 | 23.14% | 10.08% | 10.29% | 10.16% | 1.66% | 1.69% |
|  | x0.05 | 25.61% | 8.95% | 8.81% | 10.24% | 1.29% | 1.27% |
|  | x0.01 | 25.72% | 8.74% | 8.65% | 10.29% | 1.26% | 1.25% |
|  | x0.001 | 25.85% | 8.79% | 8.57% | 10.34% | 1.27% | 1.24% |
|  | x0.0001 | 25.86% | 8.82% | 8.53% | 10.34% | 1.28% | 1.23% |
| δ_X_ δ_Y_ | x10 | -12.18% | -6.95% | -7.02% | -3.85% | -0.84% | -0.85% |
|  | x0.1 | 4.96% | 4.42% | 3.75% | 2.95% | 0.83% | 0.73% |
| γ_A_ | x10 | 0.85% | 0.53% | 0.52% | 1.32% | 0.26% | 0.26% |
|  | x0.1 | -12.39% | -4.55% | -4.89% | -3.93% | -0.49% | -0.54% |
| γ_B_ | x10 | -0.16% | -0.04% | 0.22% | 0.92% | 0.17% | 0.21% |
|  | x0.1 | 1.29% | 6.62% | 5.02% | 1.49% | 1.15% | 0.92% |
| γ_C_ | x10 | -0.13% | 0.26% | -0.17% | 0.93% | 0.22% | 0.16% |
|  | x0.1 | -0.01% | 0.38% | 0.50% | 0.98% | 0.24% | 0.25% |
| K_x_ K_y_ | x10,000 | 36.31% | 3.48% | 3.59% | 14.49% | 0.49% | 0.51% |
|  | x1,000 | 36.07% | 4.62% | 4.80% | 14.39% | 0.66% | 0.68% |
|  | x100 | 35.32% | 6.21% | 6.22% | 14.10% | 0.89% | 0.89% |
|  | x50 | 34.48% | 6.92% | 6.96% | 13.76% | 1.00% | 1.00% |
|  | x10 | 28.97% | 8.90% | 9.15% | 12.48% | 1.49% | 1.52% |
|  | x0.1 | -42.74% | -23.34% | -23.89% | -15.98% | -3.25% | -3.33% |
