# Supplementary material for: Cross-Talk and Information Transfer in Mammalian and Bacterial Signaling
Source: PLoS One. 2012 Apr 18;7(4):e34488. doi: 10.1371/journal.pone.0034488 (PMC3329486; doi:10.1371/journal.pone.0034488)
Supplement: Table S6 — Effect of Asymmetric Parameter Change. (DOCX) [file pone.0034488.s016.docx]

Table S6. Effect of Asymmetric Parameter Change

|  |  | Percent Change in Information (% of Bits) | | | Absolute Change in Efficiency (%) | | |
| --- | --- | --- | --- | --- | --- | --- | --- |
|  | Fold  Change | I(X,Y;Z) | I(X;Z) | I(Y;Z) | $\frac{I\left( X,Y;Z \right)}{H\left( X,Y;Z \right)}$ | $\frac{I\left( X;Z \right)}{H\left( X;Z \right)}$ | $\frac{I\left( Y;Z \right)}{H\left( Y;Z \right)}$ |
| δ_R_ | x10 | -14.97% | -62.97% | 107.18% | -4.96% | -9.06% | 15.92% |
|  | x0.1 | 11.11% | 31.72% | -12.25% | 5.39% | 4.84% | -1.62% |
| δ_X_ | x10 | -5.22% | -24.09% | 34.04% | -1.09% | -3.36% | 5.18% |
|  | x0.1 | 2.55% | 9.95% | -2.77% | 1.99% | 1.64% | -0.23% |
| γ_A_ | x10 | 4.81% | 306.52% | -55.28% | 2.89% | 45.18% | -7.94% |
|  | x0.1 | 1.66% | -64.08% | 307.59% | 1.64% | -9.23% | 45.34% |
| γ_B_ | x10 | -0.11% | 0.19% | 0.24% | 0.94% | 0.21% | 0.22% |
|  | x0.1 | -0.14% | -0.04% | 0.26% | 0.92% | 0.17% | 0.22% |
| γ_C_ | x10 | -0.09% | 1.36% | -1.03% | 0.94% | 0.38% | 0.03% |
|  | x0.1 | 0.44% | -8.12% | 14.01% | 1.16% | -1.01% | 2.24% |
| K_x_ | x10 | 15.88% | 48.65% | -13.64% | 7.28% | 7.32% | -1.82% |
|  | x0.1 | -14.43% | -65.60% | 117.47% | -4.74% | -9.45% | 17.43% |
