# Supplementary material for: Cross-Talk and Information Transfer in Mammalian and Bacterial Signaling
Source: PLoS One. 2012 Apr 18;7(4):e34488. doi: 10.1371/journal.pone.0034488 (PMC3329486; doi:10.1371/journal.pone.0034488)
Supplement: Table S7 — Model 1 Information and Entropy. (DOCX) [file pone.0034488.s017.docx]

Table S7. Model 1 Information and Entropy

|  | I(X,Y;Z) | I(X;Z) | I(Y;Z) | I_Y_(X;Z) | I_X_(Y;Z) | I(X;Y;Z) | I(Y;X;Z) |
| --- | --- | --- | --- | --- | --- | --- | --- |
| Z= RSmad:p | 3.47 | 0.675 | 0.675 | 2.80 | 2.80 | 2.13 | 2.13 |
| Z= RSmad:Co-Smad | 3.49 | 0.675 | 0.675 | 2.82 | 2.81 | 2.14 | 2.14 |
| Z=( RSmad:p, RSmad:Co-Smad) | 3.52 | 0.680 | 0.680 | 2.83 | 2.83 | 2.15 | 2.15 |
|  |  |  |  |  |  |  |  |
|  | H(Z) | H(X) | H(Y) | H(X;Y) | H(X;Z) | H(Y;Z) | H(X,Y;Z) |
| Z= RSmad:p | 9.23 | 4.70 | 4.70 | 9.40 | 13.25 | 13.26 | 15.16 |
| Z= RSmad:Co-Smad | 11.26 | 4.70 | 4.70 | 9.40 | 15.29 | 15.29 | 17.17 |
| Z=( RSmad:p, RSmad:Co-Smad) | 8.58 | 4.70 | 4.70 | 9.40 | 12.60 | 12.60 | 14.46 |
