# Supplementary material for: Cross-Talk and Information Transfer in Mammalian and Bacterial Signaling
Source: PLoS One. 2012 Apr 18;7(4):e34488. doi: 10.1371/journal.pone.0034488 (PMC3329486; doi:10.1371/journal.pone.0034488)
Supplement: Table S8 — Parameters and Initial Values for Large Dynamic Range Two-Component Model. (DOCX) [file pone.0034488.s018.docx]

Table S8. Parameters and Initial Values for Large Dynamic Range Two-Component Model

| **Parameter** | **Standard Rate** | **Units** | **Description** |
| --- | --- | --- | --- |
| **k_X_ = k_Y_^4^** | **1E-5** | **1/(molecule*second)** | **ligand association rate** |
| **δ_X_ = δ_Y_^4^** | **5E-5** | **1/second** | **ligand dissociation rate** |
| k_ap_^3^ | 0.001 | 1/second | HK autophosphorylation |
| **k_lp_^3^** | **1** | **1/second** | **HK autophosphorylation w/ L** |
| k_ad_^3^ | 5E-4 | 1/second | HK dephosphorylation |
| **kb_11_=kb_22_^3^** | **4E-6** | **1/(molecule*second)** | **cognate RR+HK binding** |
| **kb_12_= kb_21_^4^** | **0 -4E-6 (variable)** | **1/(molecule*second)** | **non-cognate RR+HK binding** |
| **k_d_^3^** | **1E-4** | **1/second** | **RR+HK unbinding** |
| **k_pt_^3^** | **2** | **1/second** | **phosphotransfer** |
| **dphos^3^** | **1.6E-3** | **1/second** | **RRp dephosphorylation** |
| δ_L_^4^ | 5E-5 | 1/second | ligand degradation |
| L1, L2^4^ | 0 – 250^3^ | molecule | ligand 1, 2 initial amount |
| HK1, HK2^3^ | 250^3^ | molecule | HK 1, 2 initial amount |
| **RR1, RR2^3^** | **10000 each^3^** | **molecule** | **RR 1, 2 initial amount** |
