# Supplementary material for: Cross-Talk and Information Transfer in Mammalian and Bacterial Signaling
Source: PLoS One. 2012 Apr 18;7(4):e34488. doi: 10.1371/journal.pone.0034488 (PMC3329486; doi:10.1371/journal.pone.0034488)
Supplement: Text S1 — Supplementary Information and Detailed Model Development. (DOCX) [file pone.0034488.s019.docx]

Supplementary Information

Contents

[Detailed Methods 2](#_Toc314739324)

[Model Development: Adaptation to Stochastic Modeling of Smad Pathway 2](#_Toc314739325)

[Stochastic Modeling of the Two-Component System 3](#_Toc314739326)

[Model Simulation and Mean/Standard Deviation Calculation 5](#_Toc314739327)

[Information Calculation 5](#_Toc314739328)

[For information calculation based on one output: 6](#_Toc314739329)

[For information calculation based on two outputs: 6](#_Toc314739330)

[Alternative Methods of Information Calculation 7](#_Toc314739331)

[Information Calculation Verification 7](#_Toc314739332)

[Full Effect of Parameters 8](#_Toc314739333)

[Full Results from Smad Model B 10](#_Toc314739334)

[Parameter Sensitivity for Two-Component Model 11](#_Toc314739335)

[Parameter Sensitivity for Smad Model C 12](#_Toc314739336)

[Adjusted SMAD and Two-Component Models 13](#_Toc314739337)

[Increased Dynamic Range Two-Component Model 13](#_Toc314739338)

[Decreased Order of Magnitude SMAD Model 14](#_Toc314739339)

[References 15](#_Toc314739340)

# Detailed Methods

## Model Development: Adaptation to Stochastic Modeling of Smad Pathway

The Smad signaling cascade for both TGF-β and BMP pathways has been mathematically modeled by a number of research groups (Schmierer, Tournier, Bates, & Hill, 2008) (Nakabayashi & Sasaki, 2009). These groups used a deterministic method to simulate the signaling, however a stochastic method is necessary for information theory. Using the experimentally-derived kinetic parameters from Schmierer et al., and model components utilized by Nakabayashi and Sasaki, the stochastic model was developed. Schmierer et al. used an estimated nuclear volume of 1 pl and a cytoplasmic volume of 2.3 pl. These volumes were utilized to convert the necessary kinetic parameters from Nakabayashi and Sasaki from molar units to molecular units. A sample conversion from molar to molecular units is shown below in Equations S1-S3. Additionally, after preliminary investigation revealed that nuclear shuttling did not affect information transmission, this component was not included in the final model. The species used, initial values, reactions, and parameters are summarized in Table S1-2.

| $\mu=0.0018 nM^{-1}s^{-1}=\frac{0.0018}{nM s}\cdot\frac{1}{2.3pL}\cdot\frac{1 L}{1E^{-9} mol}\cdot\frac{1E^{12}pL}{1 L}\cdot\frac{1 mol}{6.022E^{23} molecules}=\frac{1.3 E^{-6}}{molecules s}$ | Equation S1 |
| --- | --- |
| $\left[ R \right]=1 nM\cdot\frac{1}{1 pL}\cdot\frac{1}{2.3pL}\cdot\frac{1E^{-9}mol}{1 L}\cdot\frac{1 L}{1E^{12}pL}\cdot\frac{6.022E^{23}molecules}{1 mol}=261 Receptors$ | Equation S2 |
| $\delta_{p}=6.6E^{-3} s^{-1}$ | Equation S3 |

Additionally, for ease of calculation, 250 receptors were chosen for each type. The amount of ligand was varied from 0 to 250 molecules because, as can be seen in Figure S1, the maximum ligand results in a plateau of maximum RC accumulation and thus incorporates the full dynamic range.

## Stochastic Modeling of the Two-Component System

Bacterial two-component systems have similarly been mathematically modeled by a number of groups. We chose to base our model on one developed by (Igoshin, Alves, & Savageau, 2008). The same process as described above was used to adapt a deterministic model into a stochastic one. The resulting reactions and parameters are tabulated in Table S3-5. The dynamic range can be seen in Figure S2.

## Model Simulation and Mean/Standard Deviation Calculation

The matrix of initial conditions is established as a 26 x 26 grid representing ligand values of 0 to 250 molecules in steps of ten for both ligand 1 and ligand 2, resulting in 676 possible initial conditions. For each of these initial conditions, 100 stochastic runs are simulated using SSC, a linux based stochastic simulation compiler (Lis, Devadas, & Chakraborty, 2009). The maximum accumulation of output was recorded for each run as averaged over a window of 50 seconds to smooth over stochastic noise. We assume that for each initial condition, the maximum accumulation of the output is normally distributed about a mean value; distributions of the data appear approximately normal. The mean and standard deviation of the maximum accumulation from the 100 runs for each initial condition are calculated and used for information calculation as described below. The output from each run through the prior is a 26 x 26 matrix of mean maximum output values, μ for each output (z or z_1_ and z_2_) as well as a 26 x 26 matrix of standard deviation values for each output (z or z_1_ and z_2_).

## Information Calculation

The standard equations for information calculation are listed below. The two forms of mutual information are given by I(X,Y,Z) in Equation S4 and I(X,Z) in Equation S5 (Reza, 1994).

| $I(X,Y,Z)=\sum_{z\in Z} \sum_{i=1}^{n} \sum_{j=1}^{m} p(x_{i},y_{j}, z)log\left( \frac{p\left( x_{i},y_{j}, z \right)}{p\left( z \right)q\left( x_{i},y_{j} \right)} \right)$ | Equation S4 |
| --- | --- |
| $I(X,Z)=\sum_{z\in Z} \sum_{j=1}^{m} p(x_{i},y_{j}, z)log\left( \frac{p\left( x_{i},y_{j}, z \right)}{p\left( z \right)q\left( x_{i},y_{j} \right)} \right)$ | Equation S5 |

Where n = number of X inputs, m = number of Y inputs. q(x_i_,y_j_) is the prior, or the probability of having a given set of initial ligand amounts. We assume an even prior so for all i and j, we have Equation S6.

| $q\left( x_{i},y_{j} \right)=\frac{1}{nm}$ | Equation S6 |
| --- | --- |

The specific probabilities are defined by Equation S7-9.

| $p\left( x_{i},y_{j},z \right)=p\left( z \vert x_{i},y_{j} \right)q\left( x_{i},y_{j} \right)$ | Equation S7 |
| --- | --- |
| $p\left( x_{i},z \right)=\sum_{j=1}^{m} p\left( z \vert x_{i},y_{j} \right)q\left( x_{i},y_{j} \right)$ | Equation S8 |
| $p\left( z \right)=\sum_{i=1}^{n} \sum_{j=1}^{m} p\left( z \vert x_{i},y_{j} \right)q\left( x_{i},y_{j} \right)$ | Equation S9 |

For information calculation, the normal distribution of output is integrated over all possible output values by binning z. Z is divided into bins that range from zero to z_max_ with a stepsize z_step_. For the ith bin, the interval of the integral is given by: $\left[ z_{i}-\frac{z_{step}}{2},z_{i}+\frac{z_{step}}{2} \right]$.

### For information calculation based on one output:

- z_max_ is set as the maximum z observed across the prior plus the maximum recorded standard deviation across the prior.
- z_step_ is set at one.
- The entire range of z from 0 to z_max_ is calculated.
- The normal cdf is taken, centered at each z value, on the interval $\left[ z_{i}-\frac{z_{step}}{2},z_{i}+\frac{z_{step}}{2} \right]$, as shown in Equation S10 below ([Mehta, Goyal et al. 2009](#_ENREF_2)).

| $P\left( z \vert x_{i},y_{j} \right)=\int_{Z-\frac{z_{step}}{2}}^{Z+\frac{z_{step}}{2}} \frac{1}{\sqrt{\left( 2\pi{\sigma_{x,y,z}}^{2} \right)}}\exp\left( -\frac{\left[ Z-\mu_{x,y,z} \right]^{2}}{2{\sigma_{x,y,z}}^{2}} \right) dZ$ | Equation S10 |
| --- | --- |

- This value is then used in Equation S7-9 to calculate the appropriate probabilities and information.

### For information calculation based on two outputs:

- z_max1_ is set as the maximum z_1_ observed across the prior plus the maximum recorded standard deviation for z_1_ across the prior.
- z_max2_ is set as the maximum z_2_ observed across the prior plus the maximum recorded standard deviation for z_2_ across the prior.
- z_step_ is set at ten.
- All possible combinations for z_1_ and z_2_ from 0 to z_max1_ and 0 to z_max2_ are calculated.
- The binormal cdf is taken, centered at each z value, on the interval $\left[ z_{1_{i}}-\frac{z_{step}}{2},z_{1_{i}}+\frac{z_{step}}{2} \right]$ and $\left[ z_{2_{j}}-\frac{z_{step}}{2},z_{2_{j}}+\frac{z_{step}}{2} \right]$ as shown in equation 8 below.

| $P\left( z_{1},z_{2} \vert x_{i},y_{j} \right)=\int_{z_{1}-\frac{z_{step}}{2}}^{z_{1}-\frac{z_{step}}{2}} \int_{z_{2}-\frac{z_{step}}{2}}^{z_{2}+\frac{z_{step}}{2}} \frac{1}{\sqrt{\left( 2\pi\sigma_{x,y,z_{1}}\sigma_{x,y,z_{2}}\sqrt{1-\rho^{2}} \right)}}\exp\left( -\frac{1}{2\left( 1-\rho^{2} \right)}\left[ \frac{\left( z_{1}-\mu_{x,y,z_{1}} \right)^{2}}{2{\sigma_{x,y,z_{1}}}^{2}}+\frac{\left( z_{2}-\mu_{x,y,z_{2}} \right)^{2}}{2{\sigma_{x,y,z_{2}}}^{2}}- \frac{2\rho\left( z_{1}-\mu_{{x,y,z}_{1}} \right)\left( z_{2}-\mu_{{x,y,z}_{2}} \right)}{\sigma_{x,y,z_{1}}\sigma_{x,y,z_{2}}} \right] \right)dz_{2}dz_{1}$ | Equation S11 |
| --- | --- |

- This value is then used in Equation S7-9 to calculate the appropriate probabilities and information.

### Alternative Methods of Information Calculation

In addition to calculating information from the summation of probabilities, one may also calculate information from the entropies. Three equations for entropy are defined below in Equation S12a-c.

| $H\left( X,Y;Z \right)=\sum_{z\in Z} \sum_{i=1}^{n} \sum_{j=1}^{m} p\left( x_{i}, y_{j}, z \right)\log\left( p\left( x_{i}, y_{j}, z \right) \right)$ | Equation S12a |
| --- | --- |
| $H\left( X;Z \right)=\sum_{z\in Z} \sum_{i=1}^{n} p\left( x_{i}, z \right)\log\left( p\left( x_{i}, z \right) \right)$ | Equation S12b |
| $H(Z) =\sum_{z\in Z} p_{Z}\left( z \right)log\left( p_{Z}\left( z \right) \right)$ | Equation S12c |

Using these entropies, one can arrive at the value for mutual information as shown in Equation S13a-b (Srinivasa, 2005).

| $I(X,Y;Z)=H(X,Y)+H(Z)-H(X,Y,Z)$ | Equation S13a |
| --- | --- |
| $I(X;Z)=H(X)+H(Z)-H(X,Z)$ | Equation S13b |

Aside from ignoring a variable in the mutual information given by I(X,Z), one may eliminate a variable by taking the conditional mutual information given by I_Y_(X,Z) in Equation S14.

| $I_{Y}\left( X;Z \right)= I\left( X,Y;Z \right)- I\left( X;Z \right)$ | Equation S14 |
| --- | --- |

The conditional mutual information allows us to calculate another value of interest, the interaction information. Interaction information, I(X;Y;Z) Equation S15, describes the amount of information (either redundancy or synergy) given by a set of variables, beyond that which is present in any subset of those variables ([McGill 1954](#_ENREF_1)). A negative value corresponds with a redundancy whereas a positive value corresponds with a synergy.

| $I\left( X;Y;Z \right)=I_{Y}\left( X;Z \right)-I\left( X;Z \right)$ | Equation S15 |
| --- | --- |

### Information Calculation Verification

Equation S1 and Equation S5 use summation of information values to determine total and mutual information. McGill provides an alternative method in the summation of entropies (McGill, 1954). These equations, given as Equation S13a, result in nearly identical values of information. The detailed calculations of this are shown in Equation S16-S18.

| $I\left( X,Y;Z \right)=H\left( X,Y \right)+H\left( Z \right)-H\left( X,Y,Z \right)=9.4+11.70-17.46=3.640 vs. 3.634$ | Equation S16 |
| --- | --- |
| $I\left( X;Z \right)=H\left( X \right)+H\left( Z \right)-H\left( X,Z \right)=4.7+11.70-15.72=0.68 vs. 0.68$ | Equation S17 |
| $I\left( Y;Z \right)=H\left( Y \right)+H\left( Z \right)-H\left( Y,Z \right)=4.7+11.70-15.72=0.68 vs. 0.68$ | Equation S18 |

# Full Effect of Parameters

The effects of all parameter changes for Smad model A are tabulated in Table S5-6. Percent change in information was calculated using Equation S19. Absolute change in efficiency was calculated using Equation S20.

| $\frac{I_{new}-I_{standard}}{I_{standard}}$ | Equation S19 |
| --- | --- |
| $\frac{I_{new}}{H}-\frac{I_{standard}}{H}$ | Equation S20 |

Note that for the large majority of parameters, there was little change in information when the parameter was varied symmetrically. Exceptions are discussed in the main article.

When parameters are varied asymmetrically, there was often a loss of information for one signal with a gain in the other. For many parameters, however, this was not a significant change in information.

To demonstrate the effect that changing various parameters has on the dynamic range of the output refer to Figures S3-4.

# Full Results from Smad Model B

To compare the information between a single output and the bivariate output, information was calculated using only RSmad:p, only RSmad:Co-Smad, and using both, as shown in Table S7.

# Parameter Sensitivity for Two-Component Model

Two key parameters were tested for their effect on information transfer over a range of cross talk values. The ligand on rate (k_x_,k_y_) and the ligand off rate (δ_x_,δ_y_) were varied over several orders of magnitude by increasing and decreasing the rates 10-fold, 1000-fold. As shown in Figure S5, the trend of a lack of robustness against cross talk is maintained throughout this range of parameter values.

The results from the parameter sensitivity for the ligand off rate (δ_x_,δ_y_) can be found in Figure S6. Similarly, we find that the lack of robustness against cross-talk appears to be insensitive to the parameter.

# Parameter Sensitivity for Smad Model C

The two key parameters identified above (Full Effect of Parameters) were tested for their effect on information transfer over a range of cross talk values. The ligand on rate (k_x_,k_y_) and the receptor internalization rate (δ_R_) were varied over several orders of magnitude by increasing and decreasing the rates 100-fold or 10-fold and 1000-fold. As shown in Figure S7, the trend of robustness against cross talk is maintained throughout this range of parameter values.

The results from the parameter sensitivity for the receptor internalization rate (δ_x_,δ_y_) can be found in Figure S8. Similarly, we find that the robustness against cross-talk appears to be insensitive to the parameter.

# Adjusted SMAD and Two-Component Models

In order to more accurately assess the effect of dynamic range on the information transfer ability, we adjusted the parameters within each model to approximately match the dynamic range of the opposite model.

## Increased Dynamic Range Two-Component Model

In order to increase the dynamic range of the two-component model to match that of the SMAD model, several parameters were adjusted. These are highlighted in Table S8. Parameters with new values are in bold. A plot of the original SMAD dynamic range and the increased two-component models is shown in Figure S9.

## Decreased Order of Magnitude SMAD Model

In order to decrease the order of magnitude of the SMAD model to match that of the two-component model, the dephosphorylation rate was increased to 50E-3 from 6.6E-3. A plot of the decreased SMAD order of magnitude and the original two-component models is shown in Figure S10.

# References

Igoshin, O. A., Alves, R., & Savageau, M. A. (2008). Hysteretic and graded responses in bacterial two-component signal transduction. *Molecular Microbiology, 68[5]*, 1196-1215.

Lis, M., Devadas, S., & Chakraborty, A. (2009). Efficient stochastic simulation of reaction-diffusion processes via direct compilation. *Bioinformatics, 25*(17), 2289-2291.

McGill, W. J. (1954). Multivariate information transmission. *Psychometrika, 19*(2), 97-116.

Mehta, P., Goyal, S., Long, T., Bassler, B. L., & Wingreen, N. S. (2009). Information processing and signal integration in bacterial quorum sensing. *Molecular Systems Biology, 5*(325).

Nakabayashi, J., & Sasaki, A. (2009). A mathematical model of the stoichiometric control of Smad complex formation in TGF-beta signal transduction pathway. *Journal of Theoretical Biology, 259*(2), 389-403.

Reza, F. (1994). *An introduction to information theory.* New York: Dover.

Schmierer, B., Tournier, A. L., Bates, P. A., & Hill, C. S. (2008). Mathematical modeling identifies Smad nucleocytoplasmic shuttling as a dynamic signal-interpreting system. *Proceedings of the National Academy of Sciences, 105*(18), 6608-6613.

Srinivasa, S. (2005). *A Review on multivariate mutual information.* Information Theory Tutorials, University of Notre Dame.
